# A Pathological Condition of the Mucous Glands of the Mouth

**Published:** 1895-02

**Authors:** Geo. Howe Winkler

**Affiliations:** NEW YORK, N. Y.


ARTICLE IV.

### A PATHOLOGICAL CONDITION OF THE MUCOUS GLANDS OF THE MOUTH.

BY GEO. HOWE WINKLER, M. D., D. D. S., NEW YORK, N. Y.

Read before the Homœopathic Medical Society of the County of New York.

When a specialist, as far removed as a dentist is from the general practice of medicine, is called upon to deliver an address before a body of general practitioners, it behooves him to carefully select from his special sphere of labor such a subject as will be both entertaining and of value to his audience. With this object in view I shall present to you this evening, from the field of stomatology, a subject that has commanded and received my most careful and studious consideration; a diseased condition that you and I meet in every day practice; that exists in nearly all of our patients, at some period of their lives, from early childhood to hoary age; that is productive of great injury to the oral cavity and of much distress to the stomach; and that is capable of successful treatment and relief without the intervention of special practice.

With this preliminary, I ask your attention to a consideration of that pathological condition of the mucous glands of the mouth, or more especially those situated on the gingival borders, or free margins of the gums, which results in the excretion of an acrid corrosive fluid that excoriates the epithelium and corrodes the teeth. I shall discuss the subject under cause, appearance, results, and treatment, and will add, as illustrations, several cases from practice. I believe this condition to be due to the elimination, by these minute glands, of hereditary poisons. All of the glands of the human body have elective affinities for such materials found in the blood as enable them to perform their normal functions. They also show a predilection for certain administered drugs; thus we prescribe, knowing that one drug will act upon the liver, another upon the kidneys, another upon the gastric glands, and so on through the entire number. They possess a still further power for good;—the power of elimination.

The elimination of injurious or poisonous products in the blood, which products may be momentary or recurring in their presence, as in the case of the salivary glands depositing the soluble salt of lime, as evidenced by the salivary calculus in the mouth, or eliminating the chlorate of potassia when administered in large doses for ulcerative stomatitis. The mucous glands of the gingival borders in a state of health secrete a clear, colorless liquid, slightly acid, which bathes the necks and crowns of the teeth, dissolving and washing away any particles of food that may lodge about them. In the exercise of their power of elimination they excrete a poisonous product which, in time, poisons the glands themselves, by its long-continued passage through them, causing severe inflammation and sloughing; and the combined products of the sloughs and eliminations work most disastrous results in the mouth, and most injurious conditions, in many instances, in the stomach.

Now these eliminations are, as I conceive, the elimination of the subtler poisons in the system, the hereditary or those of a dyscrasia, which seldom are of criminal character. The inordinate use of calomel, the excessive indulgence in liquor, tobacco, coffee, tea, etc., are sufficient causes for transmitted weakness or defects. When physicians are obliged to call a halt upon injurious indulgences in order that they may be able to restore health, or to save even life itself in some instances, it is within the bounds of reason to expect the offspring of such acquired morbid states to inherit defective constitutions. To these transmitted conditions I attribute the disease under consideration, for the reasons that it is non-syphilitic, it is not due to a want of cleanliness, it is almost universal, being found in nearly four out of five persons with whom we come in contact, the number of persons affected by it is increasing, and in the vast majority of cases it exists in patients who could by no possibility have acquired it during their own lives, either through disease, salivation, or other provocative causes.

So much for the cause. The appearance of the disease exhibits usually a line of inflammation extending along the festoons of the gums and the interdental spaces, sometimes along one or two of the molar teeth only, at other times along the palatal or lingual margins of all the teeth, and again the entire gingival borders are involved. The discoloration is sometimes so slight as to require close scrutiny to detect it; at other times the diseased border is turgid, and purple in color; again a crimson line marks the site of the affection, and in more advanced stages we have raw surfaces and phagedenic ulcerations.

The glandular excretion in some patients is clear, limpid, and sharply acid. The teeth appearing clean, but presenting slight erosions at their necks, which are of the same color as the teeth, firm to the pressure of an instrument but exquisitely sensitive; or the erosions may present the chalky white appearance of rapid acid disintegration, slimy in feeling, with pits penetrating here and there along the line of decalcification, and like the first extremely sensitive. In other cases the excretion is turbid, viscid, excoriating, corrosive, and fetid or putrid, covering the teeth with a murky or yellowish slime, which no amount of cleaning can keep off while the disease exists.

The results of this disease present a number of phases. In the first place, we have slight erosions around the teeth, which are sensitive to the touch or to sweets when taken into the mouth. Patients sometimes complain that they are not able to clean their teeth or use the pick without pain, or the erosions may be more generally distributed over the surfaces of the teeth, and in the case of little children sometimes the pain is so great that they cannot sleep except under the influence of anodynes. One case that came under my observation was a little child of two and a half years of age, who suffered so much that her mother took her to a dentist, who, not knowing how to treat the case otherwise, extracted five teeth, and left the disease untouched to continue its attacks upon the other teeth, which were already beginning to show signs of its action. In other cases the destruction of the teeth is more generally distributed, the mouth being bathed in acid and the decay presenting the light appearance of acid destruction, known to the dental profession as white decay. This condition is frequently met with in the case of young people arriving at the age of puberty, from twelve to sixteen years of age; the patients often otherwise presenting the splendid appearance of robust physical health.

I recently received a letter from a patient of mine in Denver, Col., inquiring concerning a young lady of nineteen years of age. I quote from my correspondent: "All teeth are decayed and require constant filling; cavities will sometimes come in a few weeks; they are all very sensitive and some of them are also sensitive near the gum, when touched on the outside. The dentist here says that the saliva is in an unhealthy condition, and that this is the cause of the trouble. Seventeen cavities have recently come,—that is, within two months. The dentist further says that the only thing to be done for the young lady is to crown all the roots, but advises that this should not be done until the teeth get so bad that they will not stand any more filling. She has been going to the dentist for the past three years every three or four months, so that the condition is becoming quite distressing." My correspondent adds in a postal: "The dentist says that the enamel is as thin as paper, and the teeth very soft, just like chalk."

This aptly describes the condition as frequently met with and as usually treated, except when the treatment consists in the extraction of all the the teeth and the substitution of artificial dentures. The teeth are frequently covered with a tenatious slime, and the extensive decay presents edges so friable as to be readily broken down by the pressure of the finger-nail. The cause of it is generally little understood by the profession at large, and their treatment of it is indescribably unsatisfactory. Cleaning the teeth thoroughly, detergent and stimulant mouthwashes, lime-water, prepared chalk, Phillip's milk of magnesia, etc., are among the usual local means resorted to, and internally acid phosphates and hypophosphites are prescribed, and that, oftentimes, when the presence of salivary calculus in the mouth indicates that there is a greater supply of soluble lime-salts in the blood than the system can utilize. These acid excretions render the mouth a more congenial incubator and home for those micro-organisms which are agents of tooth-destruction, and fulfill most completely the requirements for electro-chemical relations between the fillings in teeth and the walls of the cavities, which results in the breaking down or crumbling of those walls, especially the cervical, which is first and most continually covered by the acid.

I believe this diseased condition to be, in very many cases, the forerunner of that dread disease "pyorrhea alveolaris," and, while the complications existing there may not be always entirely combated, its treatment, in the beginning of operations, is imperatively demanded and invariably of benefit. These excretions, aside from destroying the teeth, are extremely distressing and injurious, giving to many patients a very disgusting taste in the mouth on waking in the morning, causing the breath to be offensive, and being swallowed with every mouthful of food, and incidentally between meals, producing occasionally distressing indigestion or dyspepsia. The treatment of this is very simple when known, but it took me a long time to discover it. For years I recognized the diseased condition and its results, and waged against it a most unsatisfactory and abortive warfare. Its successful treatment was finally accomplished through a study of the subject that was partly the outcome of an accident. I am an allopath both by my medical and dental education, and for years exhausted the resources of that school in my efforts to combat this condition. My want of success convinced me that local or topical applications could not cure the mucous glands of the mouth any more than they could cure other glands of the human body when diseased, and I became convinced that these glands, like all others, could only be reached and cured by the administration internally of such drug or drugs as would have a specific action upon them.

The paucity of internal remedies furnished by allopathy for the treatment of diseases of the mouth, and the accidental reading of a homœopathic paper, let me into a study of the homœopathic treatment of the mouth and the remedies. Among the latter I found kreosotum, which in my four years' use of it has proved itself to be almost an absolute specific for the disease we are considering, and in consequence of the great frequence of the demand for it I consider it the most valuable medicine I have at hand in the practice of dentistry. I frequently administer it with very marked benefit for several weeks before excavating decay, for filling, in very sensitive teeth. I administer it in powders, pellets, or in liquid form, from 6 x to 12 x, prescribing that form which may be most conveniently taken by each individual, in such oft-repeated doses as will secure for them the dynamic force of the drug.

In order to emphasize the above facts, I cite the following out of a great many cases that I have treated.

Case I. Mrs. J. C. C., a prominent southern lady, came to me on June 2, 1890, to have her mouth put in order. I found several cavities in her teeth, which I filled. I also discovered an extremely acid condition of the secretions, a line of corrosion around the necks of nearly all the teeth, and a sensitiveness which was almost excruciating to the touch of the tooth-brush or wooden pick, and extremely painful when sweets were taken into the mouth. I prescribed a detergent and stimulant mouth-wash, and advised that precipitated chalk be rubbed thoroughly in between and around the teeth upon retiring at night. I continued the treatment for this condition for a year, using at times lime-water and bicarbonate of-soda washes, and occasionally inserting one or two fillings; the pain that she suffered being at no time alleviated, but always most persistent and distressing.

On June 9, 1891, a year later, having discovered my present treatment, I prescribed kreosotum in pellets, which were taken for about three weeks. In a few days after beginning the medicine she was relieved of pain, and has been comfortable ever since; and the decay of the teeth has been so arrested that it was not until July 27, 1894, that she came to me again for services, requiring then but two fillings. She was a martyr to the pain and discomfort of this disease during that first year, and during the same period required the treatment and filling of several roots and of ten cavities.

Case 2. Miss S. A., between eleven and twelve years of age, was sent to me by her father, in April, 1893, just before the family went down to their summer cottage at Long Branch. I put her mouth in perfect order, finishing the work on the 27th day of April. On her return to the city, she called at my office, September 30 (I mention the dates in order to show the tremendous rapidity with which this disease works injury). I was astounded by an inspection of her mouth; it presented in the short space of five months, from a state of perfect health and condition, the appearance that is met with in some of the mulattoes of the south who suffer from strumous diathesis. The teeth were covered with a dirty-yellow, tenacious slime, the mouth reeking with acidity, and nineteen cavities of decay existing. The teeth seemed literally as if they were being

3

corroded out of her mouth. The child presented otherwise a picture of perfect physical health.

I filled fourteen cavities, and polished off four which were superficial, treating her mouth in the mean time with kreosotum 12 x, in pellets. In december, 1893, I renewed a temporary filling. On May 29, 1894, over seven months from the treatment, and just before a departure to their summer home, I examined the mouth again. There was not a cavity in it, nor a particle of slime; the teeth were perfectly clean, appearing hard and sound, and the mouth was perfectly healthy. On their return, October 5, 1894, I treated the child again, and found but four small cavities.

Case 3. Mr. W. D. W., a merchant, presented himself December, 1893, suffering with subjective pain throughout the entire maxillary region. He stated that he had been suffering for three weeks with pain, which had become so distressing that he was obliged to leave his business and come to me. On examination I discovered that his teeth were not decayed in the slightest degree, but were eroded along their necks by an acid exudation from the gingival borders. His saliva was viscid, ropy, blood streaked, and putrid. His breath was extremely fetid. I prescribed kreosotum, C. M., five powders. The pain was completely relieved in two or three days, and at the end of a week I administered kali phosph., 30 x, and one week later when I saw him his mouth was very much improved, his breath was quite pure, the saliva was almost normal, and he informed me that I had done him a great deal of good otherwise than in his mouth; he stated that for four years he had been subject to an indigestion that waked him from sleep about two o'clock in the morning, with a pain such as might be caused from a blow in the pit of the stomach, and which lasted from thirty minutes to an hour and a half, and that when he awoke in the morning he was very much nauseated until he could wash out and get rid of the ropy, putrid saliva, and disgusting taste that was in his mouth, and that since he has taken my first prescription he had been free from both nausea. I saw him a few days ago, and he had had no return ever since of that pain. His stomach had been simply poisoned by the putrid corrosive excretions of his mouth, and when I cured that condition the stomach was relieved.

Case 4. Mrs. H. D. G., about thirty years of age, the wife of a physician in the United States Marine Hospital Service, came to me June 6, 1894, from the Marine Hospital at Stapleton, S. I., and complained that her teeth ached very much whenever she ate candy, of which she was very fond. I examined her mouth and found a beautiful set of teeth, none of which were decayed, but some of the molars were slightly eroded at the necks. I prescribed kreosotum, 12 x, in pellets, and gave her an appointment a week off. She came to it and bluntly announced: "Doctor, your medicine has not done me a bit of good, and Dr. G. (her husband) laughs at Dr. Winkler's little sugar pills." I rather enjoyed the joke, inasmuch as he was just to me, by reminding her of the times for taking the medicine when she would forget. I gave her another two drachm vial of pellets, and an appointment for the following week; still her teeth were no better. I gave her another vial June 23, and urged her not to be discouraged, as I felt sure I would relieve her.

On July 16 I received a letter from her husband informing me that his wife had been quiet sick for the past two weeks, and unable to come to me, but that she had taken my medicine and had improved very much, and that she would soon be up to see me. I did not see her again, but she wrote to me from Washington, D. C., to which place her husband had been ordered, explaining their move and asking for my bill. She said further, and I quote from her letter, "We got our orders by telegraph, and of course had to leave immediately. I was awfully sorry not to be able to have you finish the work on my teeth; but you certainly did them a world of good, for they have entirely gotten over that sensitive feeling and feel in every way comfortable."

In none of these cases was any local treatment used or prescribed. I depended entirely upon the treatment of the diseased glands by kreosotum, administered internally. It is a specific for acrid corrosive secretions in the mouth. One of the peculiar exhibitions of its action in several cases in which its administration resulted quite satisfactorily was the throwing out on the face and neck of an eruption, sometimes in small pimples, and again in large blotches, which gradually faded out as the medicine was continued. One patient had a most violent itching of the the ears, beginning at about five o'clock in the afternoon and lasting for about three or four hours each afternoon with increasing intensity for three days; she then reported to me and I gave her sulfur m., and she was relieved. Now, is not this the stirring up and throwing out of some dyscrasia or occluded condition?

In conclusion, gentlemen, I would urge you to be mindful of the prevalence of this disease in the mouths of your patients, and I feel assured that you will be much gratified at the great benefit you will confer by treating the condition whenever you find it. I am honestly and thoroughly convinced that fifty per cent. of the cavities of decay which are found in most persons' teeth at the age of maturity could have been prevented; in other words, I believe that fifty per cent. of the decays of young people's teeth is absolutely preventable. And I think that no treatment for dyspepsia can be quite so efficient as that which begins in the oral cavity.—*Dental Cosmos.*

